# Can we rely on simulated patients’ satisfaction with their consultation for assessing medical students’ communication skills? A cross-sectional study

**DOI:** 10.1186/s12909-015-0508-x

**Published:** 2015-12-18

**Authors:** T. Gude, H. Grimstad, A. Holen, T. Anvik, A. Baerheim, O. B. Fasmer, P Hjortdahl, P. Vaglum

**Affiliations:** 1 Department of Behavioural Sciences in Medicine, Institute of Basic Medical Sciences, Faculty of Medicine, University of Oslo, Postbox 1111-Blindern, Oslo, N-0317 Norway; 2 Research Institute, Modum Bad, Oslo, Norway; 3 Department of Public Health and General Practice, Faculty of Medicine, Norwegian University of Science and Technology, Trondheim, Norway; 4 Department of Neuroscience, Norwegian University of Science and Technology, and St. Olav University Hospital, Pain Care Unit, Trondheim, Norway; 5 Department of Community Medicine, Faculty of Health Sciences, University of Tromsø, Tromsø, Norway; 6 Department of Global Public Health and Primary Care, Faculty of Medicine and Dentistry, University of Bergen, Bergen, Norway; 7 Department of Clinical Medicine, Section for Psychiatry, Faculty of Medicine and Dentistry, University of Bergen, Bergen, Norway; 8 Department of Family Medicine, Institute of Health and Society, Faculty of Medicine, University of Oslo, Oslo, Norway

**Keywords:** Communication skills, Simulated patients, Medical trainees, Young doctors, Medical students

## Abstract

**Background:**

In medical education, teaching methods offering intensive practice without high utilization of faculty resources are needed. We investigated whether simulated patients’ (SPs’) satisfaction with a consultation could predict professional observers’ assessment of young doctors’ communication skills.

**Methods:**

This was a comparative cross-sectional study of 62 videotaped consultations in a general practice setting with young doctors who were finishing their internship. The SPs played a female patient who had observed blood when using the toilet, which had prompted a fear of cancer. Immediately afterwards, the SP rated her level of satisfaction with the consultation, and the scores were dichotomized into satisfaction or dissatisfaction. Professional observers viewed the videotapes and assessed the doctors’ communication skills using the Arizona Communication Interview Rating Scale (ACIR). Their ratings of communication skills were dichotomized into acceptable versus unacceptable levels of competence.

**Results:**

The SPs’ satisfaction showed a predictive power of 0.74 for the observers’ assessment of the young doctors and whether they reached an acceptable level of communication skills. The SPs’ dissatisfaction had a predictive power of 0.71 for the observers’ assessment of an unacceptable communication level. The two assessment methods differed in 26 % of the consultations. When SPs felt relief about their cancer concern after the consultation, they assessed the doctors’ skills as satisfactory independent of the observers’ assessment.

**Conclusions:**

Accordance between the dichotomized SPs’ satisfaction score and communication skills assessed by observers (using the ACIR) was in the acceptable range.

These findings suggest that SPs’ satisfaction scores may provide a reliable source for assessing communication skills in educational programs for medical trainees (students and young doctors).

Awareness of the patient’s concerns seems to be of vital importance to patient satisfaction.

## Background

In the medical curriculum, teaching methods must involve faculty members to a certain degree. However, there is also a need for intensive training, for instance in clinical communication skills. The use of “lay” resources in such intensive training can be cost effective and can help save faculty resources. The use of simulated or standardized patients (SPs) in assessing medical trainees’ clinical performance, including communication skills, has been documented in research programs [1] and implemented in training programs as part of learning objectives [2]. The use of actors as SPs is convenient and can come close to simulating realistic experiences for the trainees being evaluated. In one study, 79 % of the medical trainees involved reported that their “patient” encounters were realistic [3].

Studies have shown that assessments by SPs who have been trained in evaluating medical trainees’ communication skills are valid when compared with professional observers’ ratings. Shirazi et al. found a Pearson’s correlation of 0.81 between SPs’ ratings and professional observers’ ratings, although this was in a small sample (n = 12) [4]. Boulet et al. found a similar level of accordance in a sample of doctors (n = 10) performing 10 sessions each to be evaluated [5]. In a review, Howley et al. found inconsistency in reported information in research involving SPs, especially about their gender, age, training, and context [6], which makes it important to investigate this topic further.

It is unclear to what extent SPs who have not been trained to evaluate medical trainees can rate their satisfaction with a consultation and whether their score can serve as a reliable measure of the quality of a trainee’s communication skills compared with evaluation by a professional observer. Further, it is important to identify the reasons for any lack of agreement between assessments by SPs and by professional observers.

On this background, we designed a comparative, cross-sectional study of videotaped consultations in a general practice setting. The observers of the trainees’ communication skills were faculty members experienced in using the Arizona Clinical Interview Rating Scale (ACIR) [7]. The “patients” were professional actors playing SPs who, after the consultation, completed a 10-item questionnaire about their satisfaction with the trainees’ performance [8].

Our two main research questions were:

1. How well does a SP’s rating of satisfaction with a consultation predict a medical trainee’s competence in clinical communication as assessed by a professional observer using the ACIR?
2. What are the reasons for discordance between the ratings of the same consultation between the SP and the observer?

## Methods

### Sample

Seventy-eight young doctors (still in medical training) were eligible for this study. They were among the 111 graduating students from the four Norwegian medical schools who had passed directly from medical school into their mandatory 1.5-year postgraduate internship, which they were just about to finish. The 111 were originally a part of a total of 320 graduating students in a national year cohort who had been invited to join our project. The 111 doctors had consented to participate in another study of clinical consultations 2 years earlier during the last semester of medical school [9]. Of the 78 eligible doctors invited this time, 75 consented to participate. Their task was to perform one consultation each with an SP in a general practice setting. Unfortunately, for technical and postal reasons, videotapes from 13 of the consultations were lost, thus leaving 62 consultations to constitute the core sample investigated in this study. Seventy per cent of the participants were female, and their mean age was 29.4 ± 3.4 years (range 24–46). This study was deemed exempt from requiring formal ethical approval in the country it was conducted in according to The Norwegian Social Dataservice (NSD).

### The patient role and evaluation of the consultations

In the literature, different ways of using the concepts “simulated” and “standardized” for lay people playing the role of a “patient” are found. The University of Melbourne web site uses the terms “simulated patients” and “standardized patients” interchangeably (http://medicine.unimelb.edu.au/about/employment_opportunities/simulated_patients). The web site refers to Barrows who state: “…simulated patients are trained to simulate realistic patient-clinician scenarios, and standardized patients are those who have been trained to portray the medical scenario consistently - from patient to patient, and from student to student.” [10]. We have found it most appropriate in our context to use the term simulated patients (SPs), even if they have not been trained for and used in regular teaching of medical students who, thus, are not familiar with this method.

Four professional female actors (mean age 44 years, range 38–53) were instructed in the performance of a specific patient role. They were trained together by a professional theatre instructor for a full day. The task was to play, as consistently as possible across consultations, a 43-year-old woman with the following clinical history based on a script: a few days before the consultation, she had observed blood on toilet paper and feared that this could signify bowel cancer, which had led to her mother’s death 10 years earlier. In addition, she had a complex psychosocial life situation. The four SPs played the role 19, 17, 16 and 15 times respectively (five of the video-recordings disappeared in the mail-system and eight interviews were by incident not performed).

The medical trainees were expected to spend up to 15 min on the consultation, while the mean duration was 12.0 ± 3.3 min (range 6–19). Immediately after the consultation, the SP rated her satisfaction with the trainee’s performance as if she had been a real patient. The uniformity of the SPs’ ratings was checked using a one-way analysis of variance, and no significant variation in satisfaction ratings between SPs was detected.

### Instruments

The SPs used a 10-item satisfaction questionnaire (Table 1), which was a slightly modified version of the form used by Hjortdahl et al. [8]. A total score was calculated as the mean of the 10 items, which were rated according to the following scale: 1 = totally disagree, 2 = disagree, 3 = neutral, 4 = agree, and 5 = totally agree. An internal consistency analysis yielded a Cronbach’s α of 0.88. The SPs’ total scores were dichotomized based on the value of 3.9 as the median split (skewed distribution). A score ≥4 (50 % of scores) indicated a “satisfactory” level and a score <4 an “unsatisfactory” level of competence in communication skills (Table 1).

**Table 1:** Patients’ satisfaction mean scores (SD) and differences between the POP and PON vs. “corresponding” consultations

As a patient, how did you feel about:	Mean score	Difference between POP and corresponding group	Difference between PON and corresponding group
	1 = totally disagree		
	2 = disagree		
	3 = neutral		
	4 = agree		
	5 = totally agree		
1) being accepted?	4.42 (0.56)	F = 0.01 n.s.	**F = 10.68 p = 0.002**
2) being taken seriously?	4.52 (0.53)	F = 3.00 n.s.	**F = 5.50 p = 0.023**
3) the opportunity to disclose your problems?	3.97 (0.54)	F = 0.44 n.s.	F = 2.71 n.s.
4) the physician being engaged with your problems?	4.44 (0.62)	F = 2.08 n.s.	F = 0.76 n.s.
5) the physician asking appropriate questions?	3.74 (10.14)	F = 1.99 n.s.	F = 4.21 **p = 0.045**
6) the physician giving satisfying information?	3.97 (0.83)	F = 3.00 n.s.	F = 5.10 **p = 0.028**
7) being relieved of your cancer concerns?	2.92 (0.96)	**F = 7.378 p = 0.009**	**F = 5.36 p = 0.025**
8) having spent enough time with the physician?	3.73 (0.83)	F = 0.01 n.s.	F = 0.73 n.s.
9) being cared for safely?	3.97 (0.80)	F = 3.48 n.s.	F = 2.99 n.s.
10) having this physician as your family doctor?	4.00 (0.92)	F = 2.34 n.s	F = 2.20 n.s.
Overall SPs’ score	3.96 (0.52)		

*n.s.* not significant, *POP* patient-only positive, *PON* patient-only negative

Items with a significant difference between groups are shown in bold

The medical trainees’ communication skills were assessed by a male psychiatrist (66 years) with many years of experience in teaching and evaluation using the ACIR. This observer had worked with psychologists and general practitioners (GPs) in an earlier part of this project to increase the consistency of scoring according to the ACIR manual. He watched the videotaped consultations and scored the skills on 14 items using a 1–5 scale as follows: 1 = not present at all, 2 = present to a small degree, 3 = present to some degree, 4 = mostly present and 5 = fully present. In determining the rating, the observer considered whether the trainee made use of the specific communication skill and how often and at what level of competence this skill was used (Table 2). The internal consistency of the ACIR was 0.91. In addition, one independent rater (male psychologists, age 59 years) scored 20 of the 62 videotapes, which had been drawn randomly from the sample. The interrater reliability between the observer and the independent rater was 0.70 (intraclass correlation coefficient ICC( 1.1)) [11]. Using the median split of 3.55 on the ACIR, the consultation scores were dichotomized into > 3.55 (50 % of scores) labelled “acceptable” levels and < 3.55 (50 % of scores) labelled “unacceptable” levels of communication skills (no scores ended exactly on 3.55).

**Table 2:** Mean scores (SD) on single items from the ACIR

ACIR item	1 = not present at all
	2 = present to a small degree
	3 = present to some degree
	4 = mostlypresent
	5 = present to a full degree
	Mean
1. Organization	3.56 (0.90)
2. Timeline	3.45 (0.86)
3. Transitional utterances	2.85 (1.02)
4. Open questioning	3.39 (1.12)
5. Smooth progress	3.77 (1.01)
6. Avoid repetition	4.21 (1.07)
7. Summing up	3.21 (0.73)
8. Understandable language	4.24 (0.94)
9. Documentation	3.66 (0.70)
10. Eye contact	4.23 (0.93)
11. No interruption	4.32 (0.95)
12. Response to concerns	3.03 (1.10)
13. Feedback	3.15 (0.81)
14. Additional questions	2.56 (1.15)

The two evaluation instruments were not known by the “opposite” part; i.e., the SPs were not familiar with the ACIR, and the trainees were not familiar with the SPs’ rating form.

The dichotomized ACIR scores were entered with the dichotomized SPs’ scores in a 2 × 2 cross-table (Table 3). This cross-tabulation yielded three subgroups. One was the “patient-only positive” (POP) group comprising those consultations that received an acceptable SP satisfaction score despite an unacceptable ACIR communication score (n = 8, 13 % of consultations). The second subgroup, labelled the “corresponding” group, comprised those consultations in which the SP’s evaluation agreed with the ACIR evaluation score (n = 45, 73 % of consultations). The third subgroup, labelled the “patient-only negative” (PON) group comprised those consultations in which the SP evaluated the doctor’s skills as unsatisfactory despite an acceptable ACIR score (n = 9, 14 % of consultations) (Table 3).

**Table 3:** Cross-tabulation with consultations characterized by acceptable/unacceptable ACIR scores (professional observers) against the SPs’ satisfaction/dissatisfaction scores

		Observers’ total scores		
		Acceptable	Unacceptable	
		>3.55 (median)	<3.55	
SPs’ total satisfaction scores	Satisfactory	23	8	31
	≥4.0	(corresponding positive)	(POP)	
	Unsatisfactory	9	22	31
	<4.0	(PON)	(corresponding negative)	
		32	30	62

The data included means, cross-tabulation, reliability analysis (scale), Pearson’s r, and intraclass correlations, and were analysed using SPSS 21.0.

## Results

From the cross-tabulation, the predictive power of the SPs’ satisfaction scores were calculated as 0.74 for acceptable communication skills (23 true positive/31 all positive) and 0.71 for unacceptable communication skills (22 true negative/31 all negative) (Table 3). That is, when an SP gave the trainee a score of 4 or 5, there was a 74 % probability that this score would correspond to an acceptable ACIR score (above the median of 3.55), whereas an unacceptable SP score (<4) gave a 71 % probability of corresponding to an unacceptable ACIR score (below the median).

Although the numbers in the subgroups were small, we examined the POP (n = 8) and PON (n = 9) subgroups to check whether any item was decisive for identifying the differences between the SPs’ assessments and ACIR assessments. One single item, “to be relieved of cancer concerns”, was rated significantly higher by the SPs in the POP subgroup compared with the corresponding group of consultations (n = 45) (F = 7.378, p = 0.009). Analysis of the PON consultations against the corresponding subgroups showed a significant difference on items related to taking care of patients explaining the SPs’ dissatisfaction (Table 1). These findings were consistent with a correlation matrix, in which the item to be relieved from cancer concern was the one with the lowest correlation value and the only score that was not significant when compared with the observers’ ACIR score.

## Discussion

Our main finding was an acceptable agreement between the SPs’ scores of satisfaction with the medical trainees in the consultations and the independent observers’ assessments of the same trainees’ communication skills (ACIR scores) as demonstrated by the predictive power values. This indicates that a satisfactory assessment given by an SP (an actor by profession in this study) can be useful for evaluating the effects of educational programs on medical trainees’ communication skills. This finding is supported by the fact that in only 13 % of the consultations did the SPs give a satisfactory score when the professional raters evaluated it as unacceptable. Thus, only a few trainees would receive a too-positive evaluation when assessed by an SP based on the design in this study.

Our results (predictive values) can be viewed as being consonant with findings (correlation levels) mentioned above [4, 5], even if our SPs were untrained in evaluating the trainees. With SPs’ scores not being on different levels, variation across SPs should not bias our results.

When investigating the cases that lacked agreement between the assessments, we found that those SPs who gave an acceptable score when the observers gave an unacceptable ACIR score (POP group) had seen a medical trainee who was able to relieve them of their concerns about cancer. In the cases when the SPs were negative and the observers gave an acceptable ACIR score (PON group), the reasons for this discrepancy were related to the trainees’ poor performance in providing care and mitigating the patient’s cancer concerns. This finding underscores the importance of identifying patients’ concerns in a consultation. In a literature review, Zimmermann et al. concluded that utterances of cues and concerns occur relatively seldom in medical consultations [12]. Privileging the biomedical aspects of the patient’s complaints can discourage him/her from disclosing concerns [13]. Therefore, it may be necessary for the trainee to initiate a focus upon the emotional aspects in a consultation [14] as part of a patient-centred communication style in order to ensure patient satisfaction [15]. However, such an expected relationship is not verified in all studies. Mead & Bower, in a review, found inconsistent relations between health providers’ behaviour and patient satisfaction [16]. These findings point to the fact that providers’ patient-centred behaviour can be complex to disentangle, containing a variety of attitudes.

Whether the SP’s (patient’s) satisfaction or the professional’s observations of the medical trainees’ communications skills should be viewed as the “gold” standard is, of course, debatable, especially when we advocate the importance of ensuring patient satisfaction by attending to the patient’s expressions of emotional cues and concerns. However, our design was aimed at testing the validity of the SPs’ evaluation and not that of the ACIR, and our method of analysing the data was not intended to devaluate the SPs’ scores.

One important aspect is whether SPs will evaluate their experience (satisfaction) in such a role-playing consultation in a different way from that of real patients in real consultations. Even if we do not know how well the SPS could identify with the role, our intention with the study design was to keep the SPs as close as possible to the experience of a real patient by not training or instructing them in how to evaluate the trainee’s performance. Further, for research purposes, it is more feasible and ethically acceptable to use SPs to avoid exploiting and distressing real patients in actual clinical settings.

Despite the small numbers in the POP and PON subgroups, in which the SPs’ satisfaction did not match the observers’ scoring of communication skills, the analysis of the SPs’ reasons for their evaluation of the trainees’ performance gave significant results. The ACIR scoring by independent observers and a blind rater for part of the consultations reached a level of inter-rater reliability viewed as satisfactory according to the literature [17].

One strength of this study is the nationwide representation of participants from all medical faculties in Norway. Another strength relates to the “calibration” of the SPs because the four actors were taught how to play the patient role in a consistent manner by the same professional instructor. One limitation of this study is the representativeness of the 62 attendants out of the 78 eligible doctors. However, the reduction from 78 to 75 (due to three doctors declining to participate) and then the further reduction to 62 (core sample) was considered to be random, and therefore should not have introduced any bias into the results. The representativeness of the 78 attendants in relation to the original sample of 111 graduating students from the total 1-year cohort in the country as a whole (n = 320) has been discussed elsewhere [9].

Another limitation can be the use of a single patient case, even though this woman’s history can be viewed as representative of a certain group of patients seen in the typical GP’s practice. That only female SPs were used, disguising a possible interaction effect between the trainees’ and the SPs’ gender, could also be a limitation. However, studies have found no such interaction effects [18, 19]. Although this study focused on the performance of trainees newly graduated from medical school, we presume that the situation also can be extended to the performance of trainees within medical school.

When transferring the results from research into a clinical setting, the findings should be interpreted cautiously and be viewed as indicative of relationships, but they still remain relevant to answering the research questions posed in this study.

## Conclusions

Accordance between assessments of satisfaction by SPs and evaluation of medical trainees’ use of communication skills by observers (using the ACIR) was in the acceptable range. This finding suggests that SPs’ satisfaction scores may provide a reliable source for assessing communication skills in educational programs for trainees (medical students and young doctors). Awareness of the patient’s concerns seems to be of vital importance to patient satisfaction.
